# On Some Diseases of Women Admitting of Surgical Treatment

**Published:** 1856-09

**Authors:** 


ART. VII.— *On Some Diseases of Women Admitting of Surgical Treatment.* By Isaac Baker Brown, F. R. C. S., Surgeon-Accoucheur of St. Mary's Hospital, etc., etc. Illustrated by 24 wood engravings. Philadelphia: Blanchard & Lea.

That excellent judgment which always prompts Messrs. Blanchard & Lea as to what works are needed by the profession, is not at fault here. A class of cases exist on the confines of surgery and midwifery, which may belong to either, and are consequently neglected by both. Of such are ruptured perineum, prolapse of the vagina, vesico-vaginal and recto-vaginal fistula, lacerated vagina, and encysted tumors of the vagina.

These and others find a description and treatment in Brown on the Surgical Diseases of Women. The author's search for material has been liberal and extended, and he has fitly joined it together. We would gladly devote much space and time to the critical analysis of his positions, but are obliged by the pressure of other duties to dismiss them with a simple word of commendation.

The book makes a handsome octavo of nearly 300 pages.

